# Econazole Nitrate Induces Apoptosis in MCF-7 Cells via Mitochondrial and Caspase Pathways

**Published:** 2014

**Authors:** Juan Sun, Chun-Hui Yu, Xue-Ling Zhao, Yang Wang, Shou-Gang Jiang, Xian-Feng Gong

**Affiliations:** a *School of Chemistry and Materials Science, Heilongjiang University, Harbin 150080, PR China.*; b *Key Laboratory of Forest Plant Ecology, Ministry of Education, Northeast Forestry University, Harbin 150040, PR China.*

**Keywords:** Econazole nitrate, Apoptosis, MCF-7 cell, Caspase

## Abstract

Econazole nitrate (EN), a synthetic compound, is now in use as a routine antifungal drug. EN was shown to have antitumor effect, the tumor cell killing mechanisms, however, remain unclear. In this research, the apoptosis-inducing effect of EN on MCF-7 cells was investigated. The results showed that EN inhibited the proliferation of MCF-7 cells in a time- and dose-dependent manner by MTT method and colony forming assay. MCF-7 cells treated with EN showed typical characteristics of apoptosis including the morphological changes and DNA fragmentation. Meanwhile, the loss of mitochondrial membrane potential was showed by ﬂow cytometry. In addition, western blot analysis showed that EN resulted in the decrease expression of procaspase-3, procaspase-9 and bcl-2. In conclusion, these findings suggest that EN may be an effective way for treating human breast cancer. The anti-tumor mechanisms of EN might involve mitochondrial and caspase pathways.

## Introduction

**Figure 1:**
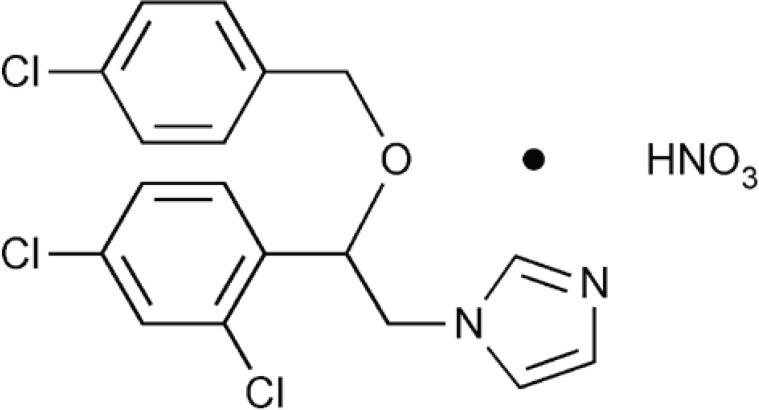
The chemical structure of econazole nitrate (EN).

It is known that azole derivatives have been shown to exert anti-tumor effect (1-4). Econazole nitrate (EN) （Figure 1）, one of imidazole anti-fungal agent (5-8), has been used in treatment of mycosis. EN has been shown to induce endoplasmic reticulum (ER) stress in HL-60 cells by promoting ER Ca^2+^ depletion and sustained protein synthesis inhibition (9-12) , suggesting that HL-60 cell is sensitive to EN. Meanwhile, EN increases the concentration of Ca^2+^ in MG63 osteosarcoma cells, which suggested that EN acts as a potent blocker of store-operated Ca^2+ ^entry (13, 14). Furthermore, EN exerts inhibitory effect on the enzymes involved in the conversion of progesterone to testosterone in the H295R cell (15). Prostate cancer PC3 cells overnight treated with econazole (1-30 μΜ) resulted in decrease in cell proliferation by 20-100% (16). EN has also been shown to induce COLO 205 cells apoptosis by arrest cell cycle and caspases signaling pathways (17).

Although some groups (15, 17) have studied the cytotoxic activity of EN against cancer cell lines, the exact mechanism remains doubtful. Meanwhile, Breast cancer is one of the most common types of cancer among women in the developed world, accounting for 15 to 20% of all cancer deaths in the mid-1990s (18), and till now little has been reported about the effect and mechanisms underlying the cytotoxic potential of EN in MCF-7 cells. Therefore, we investigated the anti-proliferative and apoptosis-inducing effects of EN in MCF-7 cells, and explore its underling mechanisms in this paper. 

## Experimental

*Material*

EN (purchased from the National institute for the Control of Pharmaceutical and Biological Products, Beijing) was dissolved in dimethyl sulphoxide (DMSO) to make a stock solution. The concentration of DMSO was kept below 3% in all cell cultures and did not exert any detectable effect on cell growth. Hoechst 33258, Rhodamine 123, MTT (3-(4, 5-dimethylthiazol-2-yl)-2, 5-diphenyltetrazolium bromide), RNase A and proteinase K were purchased from Sigma Chemical (St. Louis, MO). Western blotting antibodies against caspase-3, caspase-9 and horseradish peroxidase-conjugated secondary antibodies were purchased from Santa Cruz Biotechnology (Santa Cruz, CA), antibody against bcl-2 was purchased from Abacam (Cambridge, MA). BeyoECL Plus purchased from Beyotime Institute of Biotechnology (Haimen, Jiangsu, China).

*Cell culture*

The MCF-7 cells which obtained from Heilongjiang tumor institute were grown in RPMI 1640 medium (Hyclone, Logan, UT) supplemented with 10% heat inactivated (56 ^ₒ^C, 30 min) fetal calf serum (Hangzhou Sijiqing Biological Engineering Materials Co., *Ltd*., Zhejiang), 2 mM glutamine (Gibco, Grand Island, NY), and maintained at 37 ^ₒ^C with 5% CO_2_ in a humidified atmosphere. 

*Cell growth inhibition test*

The MTT colorimetric assay was used to evaluate the inhibition effect of EN. The cells (5×10^4^ cells per well) were seeded in a 96-well plate for 24 h, then treated with EN ranging from 0 to 120 μM for 24 h, 48 h, 72 h, respectively. After different indicated exposure times, 20μL (5 mg/mL) of MTT was added and incubated at 37℃ for 4 h .The medium was removed and the formed formazan crystal was dissolved in 150 μL of DMSO. Absorbance in control and drug-treated wells was measured at 490 nm with an ELISA reader (BioRad 680, Hercules, CA).

*Colony forming assay*

MCF-7 cells were plated in 6 wells petri dishes. The next day, the medium was changed and then various concentrations of EN（0-80 μM）was added. The incubation medium was renewed during the experiment for 14 days. At the end, cloning colony formed and putted natural dry，then colonies with more than 50 cells were counted under an inverted microscope (Olympus CKX-41, Japan).

*Morphology observation*

The cells were treated with 0 and 40 μM EN for 48 h. Then cells were washed twice with 1mL phosphate-buffered saline (PBS) and fixed in 4% paraformaldehyde for 1 h at room temperature. The fixed cells were suspended in 100 μL PBS and stained with Hoechst 33258 for 15 min at room temperature. Morphological changes of cells were observed by fluorescence microscope (Nikon TE 200-U, Japan) (19).

*Measurement of apoptosis in cells*

MCF-7 cells, containing adherent and floating, were collected by centrifugation at 1000 g for 3 min. The cell pellets were washed with PBS, and then lysated in 100 μL of lysis buffer (10 mM, Tris-HCL pH 7.4, 10 mM EDTA, pH 8.0, 0.5% Triton X-100) for 1 hour at 4 ^ₒ^C. The supernatants were incubated with 40 μg/L proteinase K and 40 μg/L RNase A at 37 ^ₒ^C each for 1 h, respectively. The DNA was precipitated with 20 μL NaCl (5M) and 120 μL isopropanol alcohol at -20 ^ₒ^C overnight, and finally centrifuged at 25000 rmp for 20 min. DNA was dissolved in TE buffer (Tris-HCl 10 mM pH 7.4, edetic acid 1 mM, pH 8.0) and separated by 2 % agarose gel electrophoresis at 100 V for 1 h. The DNA was visualized by ethidium bromide staining.

*Mitochondrial membrane potential (ΔΨm)*

Flow cytometric analysis was performed to determine mitochondrial membrane potential (20). MCF-7 cells were treated with EN 0, 20, 40, 60 µm for 48 h. The cells were centrifuged, resuspended in PBS containing 0.5 % FCS, and incubated with Rhodamine 123 (5 μΜ) in a incubator ( 37 ^ₒ^C, 5% CO_2_ ) for 40 min, then changes of mitochondrial potential were detected by flow cytometry (PARTEC, Germany).

*Western blotting*

Western blot analysis was performed as previously described (21) with some modification. Briefly, MCF-7 cells treated with different concentrations of EN for 48 h were harvested, rinsed twice with cold PBS, and resuspended in lysis buffer. Equivalent amounts of protein (30-40 µg) were separated by electrophoresis in 12% SDS polyacrylamide gel and blotted onto nitrocellulose membrane. Membranes were blocked with 5% defatted milk, and then incubated overnight with the appropriate primary antibody at dilutions specified by the manufacturer, followed by incubation at room temperature for 1 h with the corresponding horseradish peroxidase-conjugated secondary antibody at 1:500-1000 dilution in TBST. Bound secondary antibody was detected using BeyoECL Plus Enhanced Chemoluminescence Regent. 

*Statistical analysis*

Each experimental value was expressed as mean ± standard deviation (mean ± SD). The results from treated and untreated control cells were compared using the Student’s t-test to assess statistical significance. A p-value less than 0.05 were considered statistically significant.

## Results

*Inhibitory effect of EN on MCF-7 cell growth*

**Figure 2:**
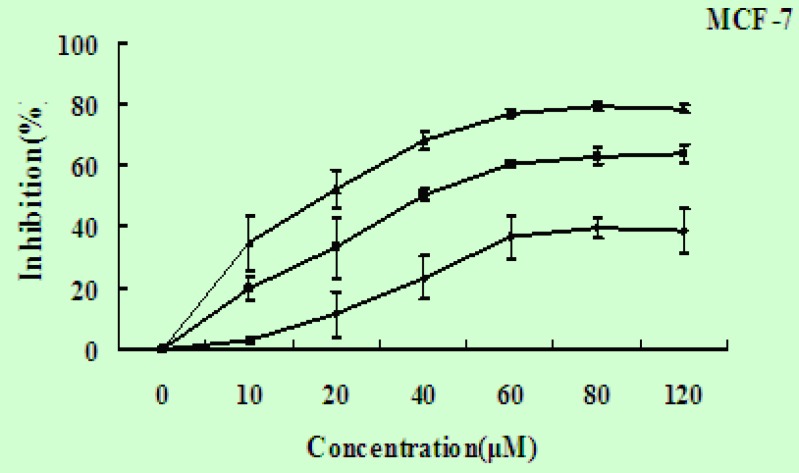
EN induces cell growth inhibition. The MCF-7 cells were exposed to EN at concentration ranging from 0-120 μM and incubated for 24 h (◆), 48 h (■) and 72 h (▲). The inhibitiory ratio was measured by MTT assay. Data represent the mean ±SD of three independent experiments

To investigate the potential effects of EN on the growth and viability of human breast carcinoma MCF-7 cells, the cells were treated with EN in varying concentrations(0-120 μM), and over varying lengths of time (24-72 h), then assessed by MTT assay. EN significantly reduced cell viability in a time- and dose-dependent manner in MCF-7 cells (Figure 2), and the IC_50_ value for MCF-7 cells is 45 μΜ for 48 h. 

*Colony forming assay effect of EN*

**Figure 3:**
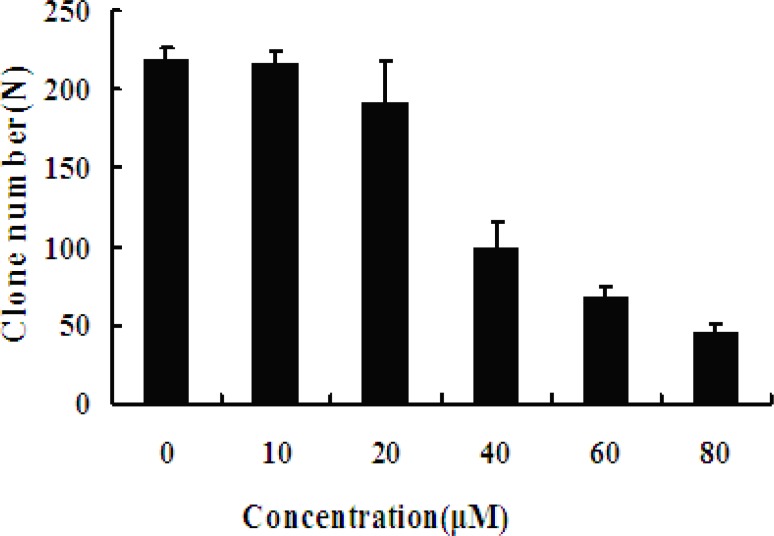
Effect of EN on MCF-7 cells colony formation. The MCF-7 cells were exposed to varied concentrations (0, 10, 20, 40, 60 and 80 μM, respectively) of EN for 48 h, and then incubated for 2 weeks totally. The cloning number was significantly decreased in treated cells compared with untreated cells. Data represent the mean ± SD of three independent experiments. ** stands for p < 0.01, and *** stands for p < 0.001, compared with the number of clones of untreated cells (0 μM).

The colony forming assay results also showed that MCF-7 cells growth is inhibited in a dose-dependent manner. This result is consistent with above result that viability of MCF-7 cells was significant influenced when exposed to EN. With the increasing concentration of EN, clone formation rate was significantly decreased (Figure 3).

*Morphological effect of EN*

The nuclear morphological changes were observed by Hoechst 33258 staining. In control group, cells were round in shape and stained homogeneously. After 24 h treatment with EN, cell markedly decreased, blebbing nuclei and granular apoptotic bodies appeared (Figure 4A). 

**Figure 4A:**
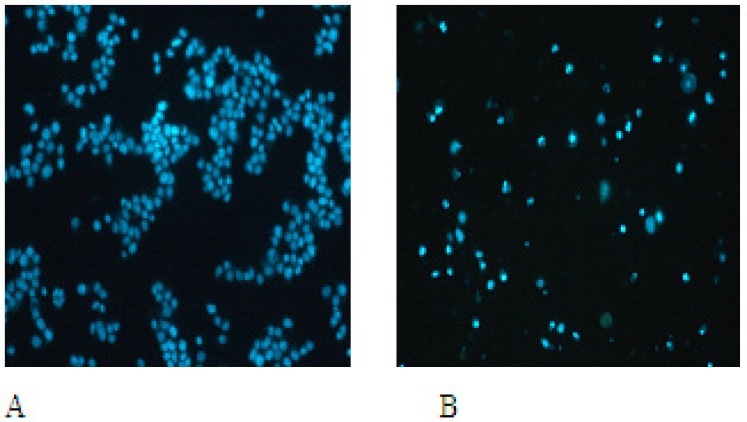
EN-induced apoptotic morphological changes. MCF-7 cells were incubated in the medium alone for 48 h (A) or in the medium containing 40  M EN for 48 h (B).

One of the specific characteristics of cell apoptosis is DNA fragmentation, which leads to the appearance of DNA ladder. The results showed that DNA ladder was obvious after 40 and 60 µM when treated with EN for 48 h (Figure 4B, left). Additionally, DNA ladder was obvious after 40 µM treatment for 48 and 72 h (Figure 4B, right), which further confirmed that EN induced cell apoptosis. 

**Figure 4B:**
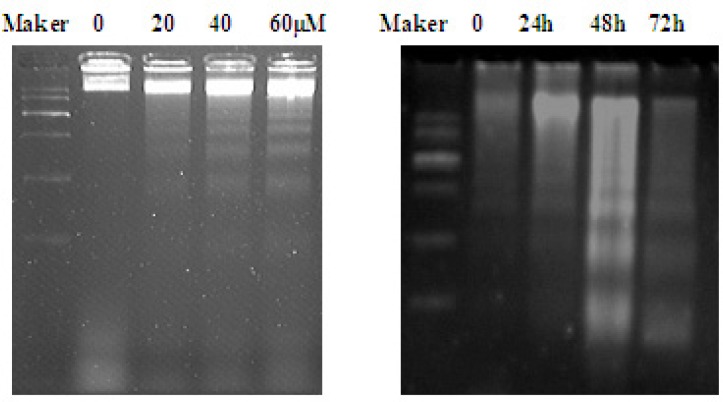
DNA fragmentation induced by EN in MCF-7 cells. The cells were treated with varied concentrations of EN (0, 20, 40 and 60 μM) for 48 h (A)or treated with 40 μM for 24 h, 48 h and 72 h (B). DNA was isolated by agarose gel electrophoresis and analyzed by ethidium bromide staining.

*Mitochondrial membrane potential effect of EN*

Mitochondria play a key role in apoptotic process. To address the possibility that EN-induced apoptosis could be related to contributions from the mitochondrial pathway, MCF-7 cells were treated with EN 20, 40 and 60 µM for 48 h. The ratio of cells with lower fluorescent intensities increases with increasing concentration of EN in a dose-dependent manner (Figure 5A), which reflected the collapse of mitochondrial membrane potential.

**Figure 5A:**
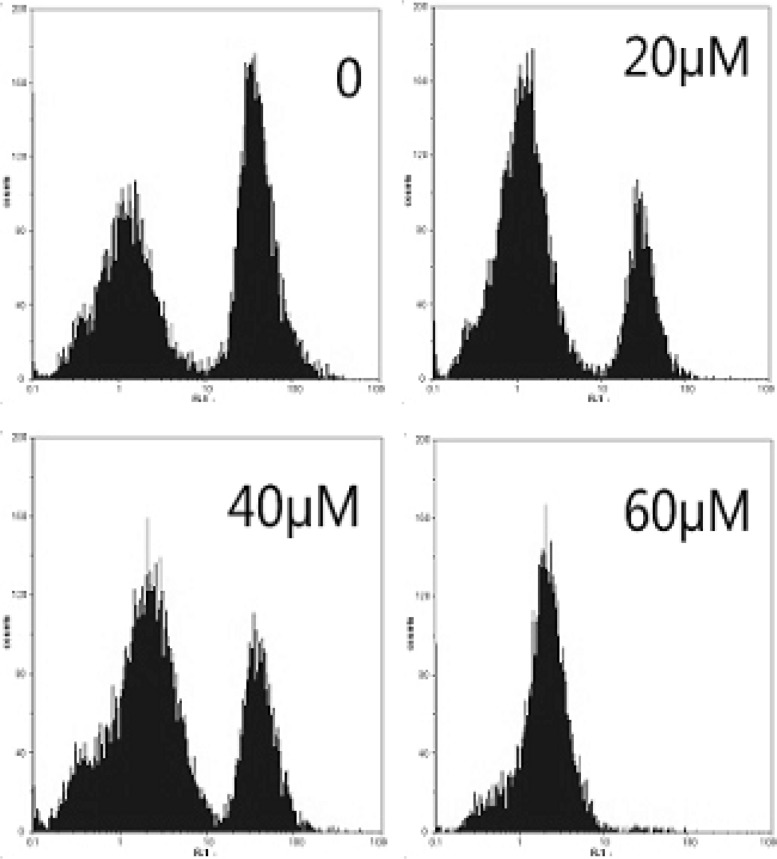
Effect of EN on mitochondrial membrane potential in MCF-7cells. MCF-7cells were treated with 0, 40 and 60 µM for 48 h, and then stained with Rhdioman 123. Changes of mitochondrial membrane potential were detected by flow cytometry.

*Western blot* *analysis of* *EN effect* *on Bcl-2 protein*

Mitochondrial Bcl-2 family is a series of proteins that regulate apoptosis and the Bcl-2 protein is an important regulator of the apoptotic cascade and promotes cell survival. We measured the expressions of Bcl-2 by western blot analysis. After incubation with EN, expression of Bcl-2 protein was decreased (Figure 5B). Based on the data, we can extrapolate that the intrinsic mitochondrial pathway may be involved in apoptosis induced by EN.

**Figure 5B:**
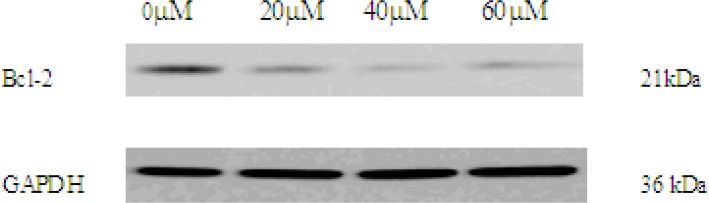
The expression of Bcl-2 in 0, 20, 40, 60 μM EN-treated MCF-7 cells. The cells were treated with EN for 48 h. Bcl-2 were analysed by western blot. GAPDH was used as an equal loading control

*Western blot* *analysis of* *EN effect* *on caspase pathway*

**Figure 6:**
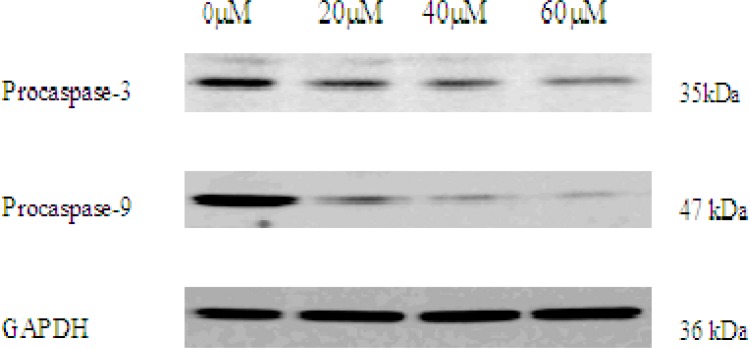
The expression of procaspase-3 and procaspase-9 in 0, 20, 40, 60 μM EN-treated MCF-7 cells. The cells were treated with EN for 48 h. Pro-caspase-3 and pro-caspase-9 were analysed by western blot. GAPDH was used as an equal loading control.

As activation of caspase-3 and caspase-9 is a hallmark of apoptotic cell death, next, we observed the expression of procaspase-3 and procaspase-9 in EN-treated MCF-7 cells by western blot analysis. In this study, after incubation with EN of indicated concentrations, expression of procaspase-3 and procaspase-9 protein was decreased, indicating caspase-3 and caspase-9 activation (Figure 6). 

## Discussion

EN is a widely used anti-fungal agent, and is also reported to possess anticancer properties (15, 17). However, little has been reported about the effects and mechanisms underlying the cytotoxic potential of EN in MCF-7 cells. In this paper, we examined the antiproliferative effect in MCF-7 cells and further studied the molecular basis of the anticancer properties of EN on the cells.

The present study showed that EN inhibited MCF-7 cell growth in a time- and dose-dependent manner. By morphological and DNA ladder assay, MCF-7 cell apoptosis induced by EN was demonstrated. 

It is well known that a family of cysteinyl proteases, caspases, is involved in the apoptotic cell death. Since caspase play very important roles in apoptotic process, involvement of caspase cascade in EN-induced MCF-7 cell apoptosis was investigated. There are at least two major pathways, initiated by caspase-8 and caspase-9, which in turn activate caspase cascade in cells. Death receptors such as Fas induce caspase-8 activation via the adapter molecule Fas-associated death domain protein (FADD). Chemotherapeutic agents and UV irradiation cause release of mitochondrial cytochrome c, which then binds the adapter molecule apoptotic proteinase activating factor-1 (Apaf-1), and this complex along with adenine nucleotides promotes caspase-9 autoactivation. Once activated, these caspases in turn activate executioner caspase, caspase-3, -6, and -7. The active executioners promote apoptosis by cleaving cellular substrates that induce the morphological and biochemical features of apoptosis (22). In our study, the expressions of procaspase-3 and procaspase-9 decreased in EN-treated MCF-7 cells, indicating activation of caspase-3 and caspase-9, which demonstrated caspases participated in this apoptotic process. Based on the above results, we presume that caspase-9 and caspase-3 played key roles in EN-induced apoptotic pathway. 

One of the major pathways for caspase activation involves the participation of mitochondria. The reduction of mitochondial membrane potential promotes release of cytochrome c (23). Bcl-2, a mitochondral protein, inhibits apoptotic process and promotes cell survival (24). Previous reports have demonstrated that Bcl-2 might directly or indirectly prevent the release of cytochrome c from mitochondrial (25, 26). Our studies showed that EN decreased mitochodrial membrane potential and Bcl-2 expression, which might suggest EN induced MCF-7 cell apoptosis by activating mitochondrial pathway. 

In conclusion, our *in-vitro* studies suggested that EN could inhibit growth of MCF-7 cells in a dose-and time-dependent manner. In addition, caspase cascade and mitochondrial pathway, including bcl-2, were involved in the apoptosis of EN-induced MCF-7 cell. Taken together, EN may be a promising chemopreventive and chemotherapeutic agent against breast carcinoma. The more detailed mechanism of EN-induced MCF-7 cell apoptosis remains to be elucidated.
